# Differences in Rehabilitation Needs after Stroke: A Similarity Analysis on the ICF Core Set for Stroke

**DOI:** 10.3390/ijerph17124291

**Published:** 2020-06-16

**Authors:** Cecilia Perin, Marta Bolis, Marco Limonta, Roberto Meroni, Katarzyna Ostasiewicz, Cesare Maria Cornaggia, Sandra Regina Alouche, Gabriela da Silva Matuti, Cesare Giuseppe Cerri, Daniele Piscitelli

**Affiliations:** 1 School of Medicine and Surgery, University of Milano Bicocca, 20126 Milano, Italy; cesaremaria.cornaggia@unimib.it (C.M.C.); cesare.cerri@unimib.it (C.G.C.); daniele.piscitelli@mcgill.ca (D.P.); 2 Casa di cura Beato Palazzolo, 24122 Bergamo, Italy; marta.bolis@casadicurapalazzolo.it; 3 Istituti Clinici Zucchi, 20841 Carate Brianza, Italy; marco.limonta@grupposandonato.it; 4 Department of Physiotherapy, LUNEX International University of Health, Exercise and Sports, Differdange, 4671 Differdange, Luxembourg; roberto.meroni@lunex-university.net; 5 Department of Statistics, Wroclaw University of Economics, 53-345 Wrocław, Poland; katarzyna.ostasiewicz@ue.wroc.pl; 6 Masters and Doctoral Programs in Physical Therapy, Universidade Cidade de São Paulo, São Paulo 03071-000, Brazil; salouche@uol.com.br; 7 Associação de Assistência à Criança Deficiente (AACD), 04027-000 São Paulo, Brazil; gmatuti@aacd.org.br; 8 School of Physical and Occupational Therapy, McGill University, Montreal, QC H3G 1Y5, Canada

**Keywords:** international classification of functioning, Disability and Health, stroke, personal factors, rehabilitation, age

## Abstract

*Background*: Successful rehabilitation is associated with physical, psychological, environmental, social, and personal factors based on the International Classification of Functioning, Disability and Health (ICF) framework. The influence of age has been suggested as crucial personal factors that may affect rehabilitation needs in post-stroke survivors. The aim of this study was to investigate the qualifiers of the ICF core set for stroke to detect differences in rehabilitation needs and goals between older (O, >65 years old) and younger (Y, ≤65 years old,) post-stroke individuals. *Materials and methods*: In this observational study, the comprehensive core set for stroke was filled during the rehabilitation period. Patient information was obtained using disability scales and translated into certain ICF categories using linking rules. Frequency, similarity, and linear regression analyses were performed for ICF qualifier profiles among Y and O patients. *Results*: Forty-eight ICF variables were significantly different between Y (*n* = 35, 46.17 ± 11.27 years old) and O (*n* = 35, 76.43 ± 6.77 years old) patients. Frequency analysis showed that activity of daily living and basic needs were more prevalent in O patients, whereas regaining of social role and social life were more prevalent in Y patients. The average Jaccard Index result (similarity analysis) was more homogeneous in O than in Y patients. *Conclusions:* ICF qualifiers are useful to design patient-centered care. Y patients have more heterogeneous needs and require more personalized program than O patients.

## 1. Introduction

Stroke is one of the leading causes of chronic disability worldwide, and persistently reduces the quality of life (QoL). Evidence suggests that rehabilitation plays a key role for improving functional status and the QoL of post-stroke individuals [1,2] as well as for other neurological disorders [3,4]. Among the factors influencing self-perceived QoL and the success of a rehabilitation program, age has been shown to be a highly significant inverse predictor of the functional outcomes (i.e., older post-stroke individuals show the worst functional outcomes) [5]. Mortality and disability after stroke are known to be more prevalent in older post-stroke individuals compared to younger [6,7,8]. In rehabilitation settings, activities of daily living (ADL) and instrumental activities of daily living (IADL) cover different needs between patients younger than 65 years. These individuals mostly tend to drop out of community activities, while older patients tend to abandon home-based activities after stroke [9,10].

Only a low proportion of older patients have access to rehabilitation, despite their high disability rate [11]. Even if they are provided advanced clinical care in an acute setting, their rehabilitative and social needs tend to be neglected [11]. These patients also showed significantly more negative attitudes about developing depression [12], i.e., depression was considered ‘inevitable and not to be treated’. In contrast, younger post-stroke individuals encounter problems with self-esteem and self–efficacy [13,14,15,16]. Younger patients reported of reduced QoL, even if they generally exhibited milder strokes [5,17,18], fragmentation in long-term care as well as lack of vocational support [19]. Younger patients frequently encounter difficulties in returning to their usual social life and to work, due to isolation, reduced fatigue tolerance [13] and the presence of many environmental barriers [14]. Nevertheless, in terms of rehabilitative outcomes, both youngers and older patients can have an acceptable personalized prognosis [9]. To this end, evidence suggests there is a mismatch between younger [15,16] and older patients [11] expectations and rehabilitation services offered.

In clinical practice, outcome measures are used to guide rehabilitation programs [20,21,22]. However, the total scores of functional measures may not completely detect individual needs [9] as well as ordinal measures may lack of responsiveness [23].

In rehabilitation, the evaluation of patients’ needs and values are crucial to develop new assessment tools, as demonstrated by the person-centered-model of care (PCC). The PCC models a system for organizing and delivering healthcare based on patients’ preferences and experiences [24] and empowering individuals to direct the course of their recovery through self-management. It is characterized as a self-management education model [25], rather than emphasizing other treating their impairments. The PCC model provides a theoretical basis for rehabilitation whereby goals can be centered on the individual and his/her lifestyle [26,27].

In the last twenty years, the most influential tool developed to describe a comprehensive perspective of health and functioning at both individual and population levels is the International Classification of Functioning, Disability and Health (ICF) [28]. The ICF includes 1424 categories, grouped in four components: “b” (body functions), “s” (body structures), “d” (activity and participation) and “e” (environmental factors). It allows the identification of individuals needs beyond their diagnoses and can help clinicians identify functional goals and treatment selection. Recently, for practical purposes, the ICF core sets were developed to link specific health conditions to salient ICF categories to guide multidisciplinary assessments. ICF core sets have been introduced to better perform needs assessment, to match interventions to specific health conditions, and to evaluate rehabilitation and outcome for each patient [29].

The ICF Core Set for Stroke [30] has been used in many studies as a framework for selection of an appropriate combination of outcome measures in broad [31,32] or specific problems [33] and a useful tool in developing comprehensive outcome measures [10,14,34,35,36,37,38,39,40] or describing patterns of disability [41]. Additionally, the ICF qualifiers specify information about the magnitude, the location and the nature of any problem of functioning. In this way, ICF qualifiers highlight strengths and weaknesses of an individual-patient and describe changes in a patient’s functional profiles over time [42].

Therefore, our study aimed to describe and compare the functioning of post-stroke individuals above and below age 65 using the ICF Core set for stroke categories and qualifiers. Specifically, qualifiers were used to assess body function impairment, activity and participation restrictions, and the role of environmental factors in younger and older patients as groups and individually.

## 2. Materials and Methods

A descriptive cross-sectional study design was used. Data were collected in two post-acute rehabilitation departments of different hospitals. The two hospitals were similar in providing health care services and medical specialties, located in comparable urban environments and serving similar patient communities. The two hospitals applied the same admission criteria for post-stroke rehabilitation.

The records of consecutive in-and outpatients over a ten-month period were collected. Inclusion criteria were: individuals who experienced a first ischemic or hemorrhagic stroke admitted for rehabilitation purposes, corresponding to International Classification of Diseases (ICD-10) codes I69.0, I69.1, and I69.3. Before the stroke participants were living at home without assistance for daily living tasks. Exclusion criteria were global aphasia (i.e., Token Test <10/36), severe cognitive impairment (i.e., Mini mental state evaluation <14/30), and age <18 years.

The following data for each participant were retrieved: sex, age, stroke etiology, time from the stroke, Functional Independence Measure (FIM) as a disability scale [21], National Institutes of Health Stroke Scale (NIHSS) as a clinical assessment tool [43], SF-36 as a quality of life questionnaire [22], Bamford Classification of clinically identifiable subgroups of cerebral infarction [44]. These evaluations were performed during the first week of hospitalization per each patient by Physical Medicine and Rehabilitation physicians. The information gathered, i.e., patient’s information and outcome measures were used to link health-status with the Comprehensive ICF Core Set for Stroke (i.e., long form [30]). Two Physical Medicine and Rehabilitation physicians, trained for the application of the ICF, translated all the information into ICF categories using linking rules [45,46,47]. For example, information about ADL, including related FIM Items and clinical examination, were translated into ICF categories belonging to d5 “Self-care”. The same two physicians used ICF qualifiers to evaluate the extent of impairments (components ”b”, “s”, “d”) and barriers and facilitators (component “e”). ICF qualifiers 0–4 were treated as an ordinal scale as specified in the ICF (0 = no influence, 1 = mild, 2 = moderate, 3 = severe, 4 = complete impairment) [28]. The qualifiers 8 (‘not specified’) and 9 (‘not applicable’) were treated respectively as missing and 0, according to most authors [29,40,48,49]. For environmental factors, the evaluation was dichotomized, distinguishing relevant (qualifier different from 0) and non-relevant (0) items. All assessments were reviewed by two senior Physical Medicine and Rehabilitation physicians with more than 20 years of experience.

The study conformed to the ethical principles of the Declaration of Helsinki. All participants signed a written consent to permit the use of their records for research purposes.

### Data Analysis

A descriptive analysis was performed to explore the demographic and clinical characteristics of the sample. Normal distribution of numeric variables was verified inspecting Q-Q plots and using Kolmogorov–Smirnov and Shapiro–Wilk tests. Frequencies and contingency tables have been used for categorical variables. The Chi-squared (χ^2^) test has been used to determine differences in proportions in contingency tables, and Fischer exact test (F) was used if one of the expected values was <5. The difference between groups (e.g., younger vs. older patients) was assessed using the Mann–Whitney (U) test when ordinal or non-normally distributed variables were involved. The Student *t*-test or analysis of variance (ANOVA) with Bonferroni correction has been used for normally distributed numeric variables.

To evaluate the similarity between ICF profiles in younger and elderly patients and to assess the distribution of the qualifiers between the two groups, statistical analysis was performed according to Goljar et al. [29] and Riberto et al. [49]. Categories in which at least 20% of the participants had some degree of a problem were selected (corresponding qualifiers had to be different from 0, 8, or 9). For these categories, frequency analysis was conducted in the sample and differences between younger (age, ≤65 years) and elderly (age, >65 years) patient groups were determined using the χ^2^ test.

In the second step of the analysis, the similarity between each pair of patients was calculated using the Jaccard index [50]. Namely, for two persons characterized by vectors: $\left(x_{1},x_{2},\ldots ,x_{N}\right)$ and $\left(y_{1},y_{2},\ldots ,y_{N}\right)$ the measure of similarity, Jaccard index, was calculated as:

(1) $$J=\frac{\sum_{i=1}^{N}\min\left(x_{i},y_{i}\right)}{\sum_{i=1}^{N}\max\left(x_{i},y_{i}\right)}.$$

We performed a comparison between mean Jaccard index of groups consisting of all possible “young” pairs and “old” pairs, considering variables related to the three components of the ICF (b, d, e) and all variables together. This analysis was repeated considering male and female subgroups since sex could be considered a confounding factor. Finally, a linear regression was performed for the Jaccard index versus the absolute value of age difference for the whole population and for both male and female subgroups, considering all the variables included in the analysis and then every component of the ICF.

The sample size for similarity analysis was computed following the recommendation proposed by Formann [51] (reviewed in [52]) that suggests a minimal sample size to include no less than 2*^k^* cases, where *k* = number of variables. The following equation was used: *k* · 2*^k^*. In our study *k* = 4, i.e., the four ICF components (“b” body functions, “s” body structures, “d” activity and participation, and “e” environmental factors). To this end, 4 × 2^4^ *=* 64. We decided to consider 10% of drop-out by possible missing data. Therefore, the final sample size (*n* = 72) was considered adequate.

Statistical analyses were performed with Statistical Package for Social Science (SPSS) v 22 (Armonk, NY, USA) and Statistical Analysis System (SAS) v 9.2 (Cary, NC, USA). Statistical significance was set at a level of 0.05.

## 3. Results

### 3.1. Study Participants

Overall, 72 records of patients who underwent rehabilitation in the selected timeframe were retrieved. Only two patients belonging to the older group were excluded due to aphasia. Therefore, data from 70 participants were considered. Thirty-five patients (10 out of 37 patients hospitalized in the first department and 25 out of 33 in the second one hospital) were younger than 65 years (46.17 ± 11.27 years old, (range: 19–63), and 35 patients were older than 65 years (76.43 ± 6.77 years old, range: 66–90). The characteristics of the study population are reported in Table 1. No significant differences between the two groups of patients were found on sex distribution, Bamford Classification, NIHSS score, and days since onset. Etiology showed a significant different distribution between the two groups. Younger patients showed higher significant days since the stroke onset compared to the older group. Older patients had significantly lower scores regarding physical activity (median, 5 vs. 20, *p* = 0.013) and general health perceptions (median, 55 vs. 70, *p* = 0.03) sections of the SF-36 (Mann–Whitney U test). No significant differences emerged from the analysis of the other sections of the SF-36.

The FIM total score was significantly higher in younger patients. Older patients were less independent in self-care, sphincter control, transfers, and locomotion, whereas younger patients encountered more problems in communication. No significant difference was found between the two groups regarding social cognition (Table 2).

### 3.2. Frequency Analysis

The qualifiers distribution showed that 85 categories were impaired in >20% of all the analyzed participants (*n* = 31 belonging to component “b”, 37 to “d” and 17 to “e”), encompassing b1 (mental functions), b2 (sensory functions and pain), b4 (functions of the cardiovascular, hematological, immunological and respiratory systems), b5 (functions of the digestive, metabolic, and endocrine systems), b7 (neuromusculoskeletal and movement-related functions), d1 (learning and applying knowledge), d2 (general tasks and demands), d3 (communication), d4 (mobility), d5 (self-care), d6(domestic life), d7 (interpersonal interactions and relationships), d8 (major life areas), d9(community, social and civic life), e1 (products and technology), e3 (support and relationships) and e4 (attitudes) categories.

When the analysis was performed with reference to age, 44 out of 85 categories revealed a similar distribution between groups, while 41 showed a statistically significant difference in the distribution between younger and older patients. Out of the 44 categories with similar distribution, those belonging to d1 (learning and applying knowledge), d2 (general tasks and demands), d4 (mobility, in particular, the use of hand, transfer, ride), d6 (domestic life), d9 (community, social and civic life), proved to be impaired in 70–90% of the records. The environmental factors e110 (products of substances for personal consumption), e310 (immediate family), e355 (health professionals), e410 (individual attitudes of immediate family members), e450 (individual attitudes of health professionals), e 460 (societal attitudes), e580 (health services, systems, and policies) were facilitators. Of the 41 ICF categories (Table 3) showing a different distribution between younger and older people, nine were more often impaired in younger patients (in italic font in the table). Younger patients seemed to demonstrate more frequently an impairment in mental functions of language (b167) and temperament and personality functions (b126).

Among the component “b”, ICF categories related to movement functions (b730, b740, b750, b770), ingestion (b510), orientation (b114), and comorbidities (b210, b410, b420) were more frequently impaired in the group of participants >65 years.

Similarly, categories of the component “activity and participation” related to motricity and activities of daily living were more often altered in older patients. Restriction of performance and capacity in communication activities and relationships was more frequent in younger patients (d310 communicating, d330 speaking, d350 conversation, and d760 family relationship).

Among the component “environmental factors” (e) more frequent facilitators for older people resulted: e115 (products and technology for personal use in daily living) (88.6%), e120 (products and technology for personal indoor and outdoor mobility and transportation) (100%) and e150 (design, construction and building products and technology for building for public use) (100%). Friends and individual attitudes of friends (e320, e420) were a facilitator mainly in younger persons (48.6% and 51.4% respectively).

### 3.3. Analysis of Similarity

Considering the entire sample and all the components analyzed (body functions, activity and participation, environmental factors), the mean Jaccard index was higher in the older group, which was more coherent (similar). The difference between the two groups was also significant (*p* < 0.05), considering every component of the ICF and only male or female patients.

The linear regression analyses showed an inverse correlation between age difference and similarity of the patients. This correlation was statistically significant, considering all variables or each component of the ICF in the whole population or in female patients alone (Table 4).

## 4. Discussion

### 4.1. General Considerations

The use of the qualifiers showed that 65% of the categories of the ICF Core Set for Stroke (85/130) represented frequent problems (more than 20% of the sample) in a population of stroke survivors admitted to two rehabilitation units. Although most of these categories (52%) presented a similar distribution in the two groups, a relevant percentage of them could identify differences between younger and older patients.

Categories related to movement, both in Body Functions (b730, b770, b740) and in Activity and Participation (d430, d450, d460, d510, d570), presented significant prevalence difference, but a very high percentage of impaired patients was observed in both groups (e.g., more than 70% in younger and more than 90% in older patients). These aspects probably represent the fundamental steps for the recovery of independence for all stroke survivors (muscle power and endurance, moving around, washing yourself, looking after one’s health).

The older group underwent a shorter period of rehabilitation and suffered from more co-pathologies. This can be related to the fact that they generally suffered from more severe stroke, as asserted by Bentsen et al. [53] and tended to slow the recovery. Moreover, in our sample older patients showed more ischemic stroke compared to younger participants that generally may lead to worse functional outcomes [54]. Knoflach et al. [5] also reported that age emerged as highly significant inverse predictor of functional outcome, Hunnicutt et al. [55] showed that in a structured training (POWER training) younger participants reached more clinically meaningful improvements than older ones. Younger patients’ compromised muscle power functions, despite better functional scores, are explained by some authors [5,56] in relation to the relevant influence of the movement abilities in working and social activities. Indeed, persistently reduced quality of life and high frequency of fatigue occur despite better rehabilitative prognosis.

### 4.2. ICF Categories Analysis

Examining the categories of the ICF (see Table 3 for ICF category codes), for which the percentage of alteration in older patients is almost twice the one in younger group, major problems concern sensations and co-pathologies (b114, b210, b410) ingestion (b510), changing and maintain body position, transferring oneself (d410, d415, d420) and basic economic transaction (d820). These results agree with Berzina et al. [39] that suggested “moving around in different locations” (d460) was negatively influenced by increasing age.

Results regarding capacities related to basic daily living, i.e., washing oneself (d510), toileting (d530) and dressing (d540) support Schnitzler et al. [57], who found that older patients were highly dependent and that individuals with stroke present more ADL problems, up to 10-fold, when compared to same-aged individuals not suffering from stroke.

In nine categories related to communication and interpersonal relationships, i.e., b126 (temperament and personality functions) and b167 (functions of language), d310 (communicating with-receiving-spoken messages), d350 (conversation) the younger group presented a significantly higher frequency of problems compared to the older group. This data cannot be explained only by the aphasic disorder, which was present in only 37% of younger patients, yet a higher percentage of patients reported difficulties in speaking and conversation (i.e., 62%). This higher percentage of difficulties reflects that younger individuals encounter problems in maintaining their family, work and social role and stresses the importance of a rehabilitation program addressing not only motor aspects but also psychological [17,58,59] and social [13,14,19] needs. However, evidence suggests that psychological and social aspects are usually missed [16,60,61,62].

Environmental factors (i.e., e115 products and technology for personal use in daily living and e120 products and technology for personal indoor and outdoor mobility and transportation) showed that older people can benefit from social aids, whereas the importance of family and social relationship as facilitators was emphasized among younger group. Indeed, the categories e115 and e120 resulted in important facilitators for older people. Problems, such as family conflict and loss of home, employment and spouse, identified in a previous study on stroke survivors under 50 years old [17] were substantially confirmed in our population: categories, like d760 (family relationships), e320 (friends) and e420 (individual attitudes of friends) were mainly altered in younger patients. Data of the present study could not cover categories related to work because most of the patients were on sick leave or on extended leave of absence.

To this end, the analysis of the body functions, activity and participation and environmental factors domains, our results, focusing on ICF qualifiers used in the framework of the Core Sets for Stroke [49,63] outlined that categories of body functions related to mobility (b7), co-pathologies (b4) and of activities related to family (d4,d5) and social life (d7,d8) were found to be the major problems in stroke survivors.

Restrictions in “activities and participation” were more typical in older patients while reporting barriers in “natural environment and human-made changes to environment”, “support and relationships” and both facilitators and barriers in “products and technology” and “attitudes” were more common among younger patients.

We confirm that the Comprehensive ICF Core Set for Stroke captures the various impairments, activity limitations and participation restrictions of individuals with stroke which are relevant to different groups and those exclusively relevant to more specific groups of patients. The frequencies of a number of impairments are distributed differently between younger and elderly groups. The difference does not necessarily signify that the two groups have different rehabilitation needs but that the most frequent complaint after stroke is toward the activities in daily living and basic needs in older patients, whereas younger patients seem to desire regaining their social role and social life. However, patients’ needs should be confirmed using specific tools in association with the ICF. We suggest that the use of the qualifiers from the ICF Core Set for stroke could be a significant contribution to define a more person centered rehabilitation program. For example, with the Goal Attainment Scale (GAS), a therapeutic method for the development of a written follow-up guide could be developed and shared with the client and family to monitor recovery [64].

### 4.3. Analysis of Similarity

The analysis of similarity (i.e., Jaccard index) revealed that the older group was more homogeneous than the younger group. This could mean that older individuals tend to have similar impairment profiles, mainly involving motor functions and activities of daily living. These findings may also suggest the possibility, for older patients, to stress those categories representing the most useful and realistic goals of rehabilitation treatment to improve its effectiveness. This could lead to a better-directed rehabilitation, a nontrivial aspect if we consider that rehabilitation in these patients is often considered poorly defined and ineffective, especially compared with social care [53]. Standardized tools with qualifiers could provide more precise measurement for research with older patients experienced stroke, who typically are not represented in randomized control trials [65]. Less similarity was found between younger patients; subsequently, defining a common profile is more difficult. The clinical consequence is that an “a priori” definition of the rehabilitation program for younger patients is not likely to be possible. Single patient-specific needs and single patient assessment is thus required. In accordance with this, Morris et al. [13] and Snögren et al. [14] highlighted that younger stroke survivors’ needs are more peculiar and different from patient to patient. The similarity analysis run for male and female showed a significant difference between the two groups, but only for females. This finding may highlight the different rehabilitation needs across sex and should be considered when developing rehabilitation programs; sex may also be considered as a prognostic factor for stroke rehabilitation [66]. Further research should examine if the ICF qualifiers might detect differences in rehabilitation needs for male and female post-stroke survivors.

### 4.4. Limits

The present work has limitations. The cross-sectional nature of this study may represent a weakness and may be not representative of the general population stroke survivors. More in-depth subgroup analysis could not be performed due to the small sample size—indeed, a better examination of variables such as ‘days since onset’ or ‘comorbidities’ is desirable. This has implications on patients profiling strategies as well, e.g., for younger people, further analysis could be performed considering different age subgroups as patients younger than 30 years, between 30 and 50 years, and older than 50 years do not necessarily present the same needs because these are different phases of life, and individuals are at different levels of self-fulfillment The sample was recruited only from two similar hospitals; a multi-center study would enhance the generalizability of our study. The linking rules to transform clinical data to the ICF categories should be investigated for reliability. Finally, due to the cross-sectional design of the study, there may be a recall bias about baseline functions prior to the stroke; however, we included only participants living at home without assistance.

## 5. Conclusions

In conclusion, our results suggest that ICF qualifiers can be considered a useful tool in detecting differences between groups of patients with the same disease. The use of the ICF Core Set and qualifiers allow an evaluation of all the problems that stroke survivors can encounter, which is not otherwise possible with a single assessment tool. This work highlights the importance of obtaining all the elements that lead to a complete assessment according to PCC model, including research on patient information, efficient training programs for chronically ill patients, and patient-centered design of care.

Future studies should investigate how information collected using the ICF Core Set should be considered when developing new treatment approaches and how ICF qualifiers might guide rehabilitation programs in clinical settings.

## Figures and Tables

**Table 1:** Characteristics of the study population and comparison between patients ≤65 and >65 years old.

Variables	Total	≤65 Years	>65 Years	Test
Sex, *n* (%)				
Male	33 (47.1)	20 (57.1)	13 (37.1)	χ^2^ = 2.809, *p* = 0.094
Female	37 (52.9)	15 (43.9)	22 (62.9)	
Etiology, *n* (%)				
Ischemic stroke	41 (58.6)	14 (40.0)	27 (77.1)	χ^2^ = 9.950, *p* = 0.002 *
Hemorrhagic stroke	29 (41.4)	21 (60.0)	8 (22.9)	
Bamford Classification, *n* (%)				
PACI	50 (71.4)	26 (74.3)	24 (68.6)	F = 0.365, *p* = 0.869
POCI	11 (15.7)	5 (14.3)	6 (17.1)	
TACI	9 (12.9)	4 (11.4)	5 (14.3)	
NIHSS (median)	5.5	6	5	U = 616, *p* = 0.967
Days since onset (mean [SD])	247.49 (471.340)	341.49 (614.382)	143.49 (226.102)	T = 1.880, *p* = 0.067

Abbreviations: PACI, Partial anterior circulation infarcts; POCI, Posterior circulation infarcts; TACI, Total anterior circulation infarcts; *p*, *p*-value; *, significant *p*-value.

**Table 2:** FIM scores in two groups of patients younger and older than 65 years.

FIM (Mean [SD])	Age ≤ 65 Years Old	Age > 65 Years Old	ANOVA (*p*)
Self-care	25.71 (10.532)	16.06 (5.657)	<0.001 *
Sphincter control	11.94 (3.686)	7.49 (4.217)	<0.001 *
Transfers	12.06 (5.765)	6.94 (3.963)	<0.001 *
Locomotion	6.74 (4.068)	3.66 (2.114)	<0.001 *
Communication	11.4 (3.283)	13.11 (1.694)	0.008 *
Social cognition	17.11 (3.297)	16.74 (2.381)	0.591
FIM (total score)	84.97 (23.872)	65.03 (18.176)	<0.001 *

Abbreviations: *p*, *p*-value; FIM, Functional Independence Measure; *, significant *p*-value.

**Table 3:** ICF variables (*n* = 41) in which older and younger patients’ groups demonstrated a significant difference in the Chi-squared test (**χ^2^**). Percentages of participants presenting an alteration are reported.

ICF Category	Tot *n* = 70	<65 y *n* = 35	>65 y *n* = 35	χ^2^	*p*
*b126 temperament and personality functions*	37.1	51.4	22.9	6.119	0.013 *
*b167 mental functions of language*	30	45.7	14.30	8.231	0.004 *
*d310 communicating with-receiving-spoken messages (capacity)*	25.7	40	11.4	7.479	0.006 *
*d330 speaking (performance)*	50	62.9	37.1	4.629	0.031 *
*d350 conversation (performance)*	35.7	48.6	22.9	5.04	0.025 *
*d350 conversation (capacity)*	51.4	68.6	34.3	8.235	0.004 *
*d760 family relationships (capacity)*	25.7	37.1	14.3	4.786	0.029 *
*e320 friends*	35.7	48.6	22.9	5.04	0.025 *
*e420 individual attitudes of friends*	38.6	51.4	25.7	4.884	0.027 *
b114 orientation functions	30	14.3	45.7	8.231	0.004 *
b210 seeing functions	54.3	37.1	71.4	8.289	0.004 *
b410 heart functions	35.7	17.1	54.3	10.516	0.001 *
b420 blood pressure functions	71.4	51.4	91.4	13.72	<0.001 *
b510 ingestion functions	37.1	17.1	57.1	11.993	0.001 *
b730 muscle power functions	94.3	88.6	100	4.242	0.039 *
b740 muscle endurance functions	85.7	71.4	100	11.667	0.001 *
b750 motor reflex functions	68.6	54.3	82.9	6.629	0.01 *
b770 gait pattern functions	94.3	88.6	100	4.242	0.039 *
d360 using communication devices and techniques (performance)	64.3	48.6	80	8.811	0.003 *
d360 using communication devices and techniques (capacity)	72.9	60	85.7	7	0.008 *
d410 changing basic body position (performance)	41.4	20	62.9	13.246	<0.001 *
d410 changing basic body position (capacity)	80	68.6	91.4	5.714	0.017 *
d415 maintaining a body position (performance)	45.7	28.6	62.9	8.289	0.004 *
d420 transferring oneself (performance)	42.3	20	65.7	14.993	<0.001 *
d420 transferring oneself (capacity)	82.3	71.4	94.3	6.437	0.011 *
d430 lifting and carrying objects (performance)	67.1	51.4	82.9	7.835	0.005 *
d430 lifting and carrying objects (capacity)	90	82.6	97.1	3.968	0.046 *
d450 walking (performance)	87.1	77.1	97.1	6.248	0.012 *
d450 walking (capacity)	94.3	88.6	100	4.242	0.039 *
d460 moving around in different locations (performance)	57.1	42.9	71.4	5.833	0.016 *
d460 moving around in different locations (capacity)	92.9	85.7	100	5.385	0.02 *
d510 washing oneself (capacity)	92.9	85.7	100	5.385	0.02 *
d530 toileting (capacity)	80	68.6	91.4	5.714	0.017 *
d540 dressing (capacity)	90	82.9	97.1	3.968	0.046 *
d550 eating (capacity)	82.6	71.4	94.3	6.437	0.011 *
d570 looking after one’s health (capacity)	92.9	85.7	100	5.385	0.02 *
d860 basic economic transactions (performance)	34.3	17.1	51.4	9.13	0.003 *
d860 basic economic transactions (capacity)	71.4	57.1	85.7	7	0.008 *
e115 products and technology for personal use in daily living	68.6	48.6	88.6	12.992	<0.001 *
e120 products and technology for personal indoor and outdoor mobility and transportation	92.9	85.7	100	5.385	0.02 *
e150 design, construction and building products and technology of buildings for public use	92.9	85.7	100	5.385	0.02 *

Abbreviations: Tot, total; y, years old; *, significant *p*-value.

**Table 4:** Analysis of similarity.

**WHOLE SAMPLE**				
	**Body Functions**	**Activity and Participation**	**Environmental**	**All Variables**
**Mean Jaccard Indexes**				
Younger-Younger	0.240324	0.371892	0.854377	0.507685
Older-Older	0.318024	0.541769	0.904944	0.614161
***p*-value for differences of average Jaccard Indexes**				
Younger vs. Older	0	0	0	0
**Linear regression (Jaccard index vs. age difference)**				
r	−0.10657	−0.10254	−0.05322	−0.10969
*p*-value	1.52616 · 10^−7^	4.42287 · 10^−7^	0.0089	6.52583 · 10^−8^
**MALE**				
	**Body Functions**	**Activity and Participation**	**Environmental**	**All Variables**
**Mean Jaccard indexes**				
Younger-Younger	0.224391	0.359342	0.850252	0.493222
Older-Older	0.318226	0.526554	0.897833	0.621498
***p*-value for differences of average Jaccard indexes**				
Younger vs. Older	1.03206 · 10^−12^	0	3.25907 · 10^−10^	0
**Linear regression (Jaccard index vs. age difference)**				
r	−0.00835	0.085099	0.018771	0.075016
*p*-value	0.848197	0.0506608	0.666941	0.0850561
**FEMALE**				
	**Body Functions**	**Activity and Participation**	**Environmental**	**All Variables**
**Mean Jaccard indexes**				
Younger-Younger	0.271427	0.381075	0.864418	0.525186
Older-Older	0.322715	0.562629	0.910483	0.618968
***p*-value for differences of average Jaccard indexes**				
Younger vs. Older	0.000081585	0	1.47882 · 10^−13^	2.22045 · 10^−16^
**Linear regression (Jaccard index vs. age difference)**				
r	−0.21674	−0.29398	−0.16131	−0.30252
*p*-value	1.60194 · 10^−8^	9.76996 · 10^−15^	0.0000288387	1.33227 · 10^−15^
